# Analysis of the traits of deliberate substance abuse and its correlation with psychological states in adolescents

**DOI:** 10.3389/fpsyt.2025.1677033

**Published:** 2026-01-09

**Authors:** Xinhui Liu, Zhihua Wang, Hongyu Liang, Xin Qi, Lihua Wang

**Affiliations:** Baoding Hospital, Beijing Children’s Hospital Affiliated to Capital Medical University, Baoding, Hebei, China

**Keywords:** adolescence, deliberate substance abuse, psychological states, risky behaviors, self-harm

## Abstract

**Background:**

Deliberate substance abuse among adolescents represents a significant public health concern, yet comprehensive analysis of its characteristics and psychological correlates remains limited.

**Objective:**

To analyze the characteristics of deliberate substance abuse among adolescents and its correlation with psychological states.

**Methods:**

This retrospective cohort study included 158 adolescents (mean age: 12.87 ± 1.32 years; 123 girls, 35 boys) admitted for deliberate substance abuse between January 2020 and December 2022. Data were collected using standardized instruments including the Risky Behavior Questionnaire for Adolescents (RBQ-A), Center for Epidemiologic Studies Depression Scale (CES-D), Multidimensional Anxiety Scale for Children (MASC), Cognitive Emotion Regulation Questionnaire-Chinese Version (CERQ-C), Adolescent Self-Rating Life Events Checklist (ASLEC), and Adolescent Social Support Scale (ASSS).

**Results:**

The number of cases increased annually (2020: 34; 2021: 54; 2022: 70), with a higher prevalence among girls than boys. The most commonly abused substances were antipsychotic medications. Univariate analysis revealed significant associations between deliberate substance abuse and factors including presence of medical personnel in the family, family structure, guardian’s education background, residential area, sole child status, knowledge of pharmaceutical security, depression/anxiety, CERQ-C scores, adverse experiences, and social support (all P < 0.05). Appropriate correlation analyses revealed that psychological states were significantly correlated with these factors (all P < 0.05). Multivariate linear regression identified guardian’s education background, knowledge of pharmaceutical security, depression/anxiety, CERQ-C scores, and social support as the primary factors affecting psychological states (all P < 0.05).

**Conclusion:**

Deliberate substance abuse among adolescents is associated with multiple sociodemographic and psychological factors. Interventions targeting modifiable factors such as drug safety education and social support may help prevent this behavior.

## Introduction

Upon entering adolescence, individuals undergo notable psychological and physiological transformations, engendering various confusions that predispose them to anxiety, depression, and, in severe instances, self-harm and suicidal behaviors (1, 2). Adolescents are undergoing a period of physical and mental growth, and are mostly mentally immature. They experience intricate shifts in their worldview and life perspectives, rendering them vulnerable to external influences. Research indicates that approximately 24.6% of teenagers grapple with uneasiness or melancholy (3). Deliberate substance abuse represents a significant public health concern among adolescents, often serving as a method of self-harm or suicide attempt. Previous research has shown that about 22.37% of Chinese adolescents engage in self-harm during adolescence, with substance overdose being a prevalent method (4). In China, there remains a critical shortfall in attention towards adolescent deliberate substance abuse, particularly regarding its correlation with psychological states. Adolescents receiving treatment for deliberate substance abuse represent a unique population with specific clinical characteristics and needs. Understanding the traits of this behavior and its correlation with psychological states in this clinical population is essential for developing targeted interventions (5). This study aims to describe the sociodemographic and clinical characteristics of adolescents with deliberate substance abuse; identify psychological and social factors associated with these behaviors; and analyze the combined influence of these factors on psychological state using multivariate modeling.

## Materials and methods

### Study design and population

This descriptive cross-sectional study was conducted using retrospective clinical data from adolescents treated for deliberate substance abuse. The data collection instruments assessed both current psychological states and historical information about substance abuse incidents documented in medical records. 158 adolescent participants who were admitted to the Department of Emergency at Baoding Hospital, Beijing Children’s Hospital Affiliated to Capital Medical University from January 2020 to December 2022. All eligible adolescents admitted for deliberate substance abuse during this period were included, representing the total population during this timeframe. This cohort ranged in age from 10 to 17 years (mean: 12.87 ± 1.32), comprising 35 boys and 123 girls. The inclusion of participants aged 10–12 years was justified as this age range represents early adolescence. The ethical committee of the hospital (approval no.: 202320; approval date: March 10, 2023) approved the study, and all adolescents and their guardians provided signed consent forms.

Inclusion criteria: (1) a clear history of drug intake; (2) detection of drug or particular enzyme-related indicator anomalies, like in the patient’s stomach contents or during blood work, urinary or fecal examinations; (3) steady psychological and bodily state, exhibiting lucidity, without cognitive or speech impairments throughout interrogations.

### Operational definition

Deliberate substance abuse was operationalized through documented evidence of intentional substance ingestion from medical records, including patient reports, laboratory findings, and clinical assessments. Medication history and details were obtained through medical record review and patient interviews conducted during hospitalization.

As this study aimed to include all eligible cases during the specified period, a convenience sample of 158 participants was obtained. *Post-hoc* power analysis indicated that this sample size provided >80% power to detect medium effect sizes (Cohen’s f² = 0.15) in multiple regression models with up to 10 predictors at α = 0.05.

### Investigation methods

Data were collected between January 2020 and December 2022. All adolescents admitted for deliberate substance abuse during this period were eligible for inclusion. Participants were approached during their hospitalization once they were medically stable and able to provide informed assent. Questionnaires were administered in a private room within the emergency department or pediatric ward by trained investigators.(1) The assessment of adolescent risky behaviors was conducted using the Risky Behavior Questionnaire for Teenagers (RBQ-A) (6), which delineates five major risk behaviors: aggression and violence, disciplinary and legal violations, smoking and drinking, suicidal and self-injurious behaviors, and unhealthy dietary habits and insufficient physical activity. This instrument employs a 5-point scoring system, where increased total scores imply elevated degrees of risky behaviors. The Cronbach’s alpha value of the scale utilized in this research was 0.81, indicating robust reliability. Previous validation studies have also demonstrated good construct validity for the RBQ-A through factor analysis and convergent validity with related behavioral measures. (2) The severity of melancholy among the youngsters was evaluated using the Center for Epidemiologic Studies Melancholy Scale (CES-D) (7). The assessment standards include melancholic affect, social connections, somatic complaints and favorable affect, comprising 20 evaluation questions with four levels each, totaling 60 points. Scores ranging from 0 to 16 denote an absence of melancholy, whereas scores exceeding 17 signal its presence. In the present study, Cronbach’s alpha for this scale was 0.90.(3) The Multidimensional Anxiety Scale for Children(MASC) scale was utilized to assess individual uneasiness levels over the preceding week (8), utilizing a 0–3 grading scale across domains including somatic complaints, interpersonal discomfort, isolation distress, and evasion of harm. The total scores indicate the severity of uneasiness symptoms; scores >60 indicate significant overall uneasiness symptoms, and >75 indicate very severe overall uneasiness symptoms. In the present study, Cronbach’s alpha for this scale was 0.91. (4) The Cognitive Emotion Regulation Questionnaire-Chinese Version(CERQ-C) assesses individuals’ mental adaptation techniques following adverse experiences (9), with 36 questions covering 9 factors: self-blame, acceptance, rumination, positive refocusing, refocus on planning, positive reappraisal, putting into perspective, catastrophizing, and other-blame, every factor comprising 4 questions with a scale of 5 points. Higher scores indicate increased utilization of a particular intellectual strategy. In the present study, Cronbach’s alpha for this scale was 0.81. (5) The Adolescent Self-Rating Life Events Checklist(ASLEC) scales examines how often and the level of stress associated with adverse experiences encountered by teenagers within the last year (10). It constitutes a prevalent self-evaluation tool for stress events in teenagers’ life, with 27 questions covering six aspects: social connections, study stress, penalty, feeling of absence, healthy adaptation, and other factors. Scoring is based on the impact level of the event on the individual, with elevated scores suggesting greater intensity of negative encounters. In the present study, Cronbach’s alpha for this scale was 0.82. (6) The Adolescent Social Support Scale(ASSS) comprises 18 questions across three domains: personal and extrinsic support (11), and utilization of aid, assessed through a scale of 5 points. Increased values signify higher social support received, primarily assessing teenagers’ community backing and favorable recognition from other people. In the present study, Cronbach’s alpha for this scale was 0.91.

### Variable categorization criteria

The following criteria were used for dichotomizing continuous variables:Guardian’s education background: “Low” = below high school; “High” = high school or above;Knowledge of pharmaceutical security: assessed via a 5-item scale; scores ≤3 = “Weak,” ≥4 = “Strong”;CERQ-C scores: median split used for “Weak” vs. “Strong”;Adverse experiences: ASLEC total score >75th percentile = “Severe”;Social support: ASSS total score < median = “Weak”.

### Quality control

The survey was conducted by uniformly trained investigators who explained the questionnaire’s structure and completion guidelines for the patients. Following the acquisition of informed consent, the patients filled out the questionnaires on the spot, and the questionnaires were collected immediately. Data entry adhered stringently to the verbatim responses, with a secondary entry verification for data consistency. Discrepancies were rectified by consulting the original questionnaires using their identification numbers, and questionnaires exhibiting an absence rate surpassing 5% or displaying excessively uniform replies were disqualified.

### Statistical analysis

The entire dataset was processed and statistically analyzed with SPSS 22.0 (SPSS Inc., Chicago, IL, USA). Count-based data were represented as percentages (%), and compared utilizing the chi-square (χ^2^) test. Measured data were represented as mean ± standard deviation (`x ± s), and compared utilizing the independent samples *t*-test. Point-biserial correlation was used for binary variables, Spearman correlation for ordinal variables, and Pearson correlation for continuous variables. Multiple linear regression was applied to multivariate analysis.The regression model’s assumptions were checked including linearity, homoscedasticity, and normality of residuals. Variance inflation factors (VIF) were calculated to check for multicollinearity. Effect sizes (Cohen’s d) were calculated for t-tests. A significance level α set at 0.05.

## Results

Between 2020 and 2022, 158 adolescents with deliberate substance abuse were treated, showing an increasing trend (2020: 34; 2021: 54; 2022: 70 cases).The demographic and clinical characteristics of the sample are presented in **Table 1**. Briefly, the sample comprised 35 boys (22.2%) and 123 girls (77.8%), with a mean age of 12.87 ± 1.32 years. The number of individuals experiencing, psychological difficulties (depression, anxiety, defiance, etc.) also increased annually (13, 20, 28 cases, respectively), with antipsychotic medications being the predominant substances ingested. Antipsychotic medications were the predominant substances ingested, including sertraline (27 cases), estazolam (15), flu medications (14), lorazepam (11), pesticides (11), rodenticides (3), zopiclone (3), and cephalosporin antibiotics (3). Clinically, the adolescents predominantly presented with digestive issues like stomach discomfort, nausea, and vomiting, while others exhibited dizziness, sleepiness, and weariness. Apart from one individual who was transferred to Beijing Children’s Hospital due to paraquat poisoning, the remaining participants showed improvement and were discharged after gastric lavage and induced excretion, with no deaths documented.

**Table 1:** Demographic and clinical characteristics of the study sample (N = 158).

Characteristic	Category	n (%) or $\left(\bar{x}\pm \text{s}\right)$
Age (years)		12.87 ± 1.32
Gender	Male	35 (22.15%)
	Female	123 (77.85%)
Family Structure	Two-parent	87 (55.06%)
	Single-parent	71 (44.94%)
Guardian’s Education	High (High school or above)	88 (55.70%)
	Low (Below high school)	70 (44.30%)
Residential Area	Urban	83 (52.53%)
	Rural	75 (47.47%)
Sole Child	Yes	95 (60.13%)
	No	63 (39.87%)
Medical Personnel in Family	Yes	42 (26.58%)
	No	116 (73.42%)
Knowledge of Pharmaceutical Security	Strong	50 (31.65%)
	Weak	108 (68.35%)
Depression/Anxiety (CES-D/MASC)	Present	61 (38.61%)
	Absent	97 (61.39%)

Univariate examination of risky behaviors and psychological states related to deliberate substance abuse in adolescents revealed statistically significant differences in several factors: presence of medical personnel in the family, family structure, guardian’s education background, residential area, sole child status, knowledge of pharmaceutical security, melancholy/uneasiness, CERQ-C scores, adverse experiences, and social support (all *P* < 0.05), as shown in **Table 2**. Effect sizes (Cohen’s d) are reported in **Table 1** to indicate the practical importance of findings.

**Table 2:** Univariate examination of risky behaviors associated with deliberate substance abuse in adolescents.

Item		n	RBQ-A scores	t	P	Effect size
Presence of medical personnel in the family	Yes	42	59.57 ± 5.67	2.988	0.003	0.55
	No	116	62.97 ± 6.52			
Family structure	Single-parent	71	63.41 ± 6.16	2.398	0.018	0.38
	Two-parent	87	60.97 ± 6.54			
Guardian’s education background	Low	70	64.11 ± 6.41	3.697	0.000	0.59
	High	88	60.43 ± 6.06			
Residential area	Rural	75	63.29 ± 6.40	2.304	0.023	0.37
	Urban	83	60.95 ± 6.36			
Sole child status	Yes	95	63.01 ± 6.25	2.292	0.023	0.37
	No	63	60.63 ± 6.57			
Knowledge of pharmaceutical security	Weak	108	63.39 ± 6.42	3.961	0.000	0.63
	Strong	50	59.20 ± 5.62			
Melancholy/uneasiness	Yes	61	66.18 ± 5.34	7.340	0.000	1.17
	No	97	59.47 ± 5.74			
CERQ-C scores	Weak	100	63.49 ± 6.07	3.795	0.000	1.60
	Strong	58	59.60 ± 6.43			
Adverse experiences	Severe	58	65.74 ± 5.32	6.028	0.000	0.96
	Not severe	100	59.93 ± 6.12			
Community backing	Weak	80	66.58 ± 4.57	12.559	0.000	1.98
	Strong	78	57.44 ± 4.57			

Appropriate correlation analyses revealed that psychological states were significantly correlated with the presence of medical personnel in the family, family structure, guardian’s education background, residential area, sole child status, knowledge of pharmaceutical security, depression/anxiety, CERQ-C scores, adverse experiences, and social support (all *P* < 0.05), as shown in **Table 3**.

**Table 3:** Correlation analysis of risk factors/behaviors and psychological states related to deliberate substance abuse in adolescents.

	Presence of medical personnel	Family structure	Guardian’s education	Residential area	Sole child status	Knowledge of pharmaceutical security	Depression/anxiety	CERQ-C scores	Adverse experiences	Social support
*r*/ρ	-0.233	-0.189	-0.284	-0.181	-0.181	-0.302	-0.507	-0.291	-0.435	-0.709
*P*	0.003	0.018	0.000	0.023	0.023	0.000	0.000	0.000	0.000	0.000

Point-biserial correlation used for binary variables, Spearman correlation for ordinal variables.

Taking psychological states (RBQ-A total score) as the dependent variable and the presence of medical personnel in the family, family structure, guardian’s education background, residential area, sole child status, knowledge of pharmaceutical security, melancholy/uneasiness, CERQ-C scores, adverse experiences, and social support as independent variables, a multivariate linear regression analysis was conducted. This analytical approach revealed that guardian’s education background, knowledge of pharmaceutical security, melancholy/uneasiness, CERQ-C scores, and community backing were the main variables affecting the psychological states of teenagers engaging in deliberate substance abuse (all *P* < 0.05), as shown in **Table 4**. The model demonstrated good fit (R² = 0.58, Adjusted R² = 0.55) and no evidence of multicollinearity (VIF range: 1.2-2.1).

**Table 4:** Multivariate analysis of factors affecting psychological states of deliberate substance abuse.

Factor	Partial regression coefficient	Standard error	Standard regression coefficient	t	P value	95%CI	VIF
Guardian’s education background	-2.188	0.778	-0.169	-2.812	0.006	-3.726 ~ -0.650	1.8
Knowledge of pharmaceutical security	-2.249	0.758	-0.162	-2.966	0.004	-3.747 ~ -0.750	1.7
Depression/anxiety	-2.426	0.794	-0.183	-3.057	0.003	-3.994 ~ -0.857	2.1
CERQ-C scores	-1.693	0.713	-0.127	-2.375	0.019	-3.102 ~ -0.284	1.5
Social support	-8.025	0.947	-0.545	-7.415	0.000	-8.898 ~ -5.153	1.2

## Discussion

Our study aimed to characterize deliberate substance abuse among adolescents and identify associated psychological factors. Several key findings emerged from our analysis. Deliberate substance abuse is among the most frequent types of non-medical self-harm and attempted suicide among teenagers globally (12). Current research indicates an increasing trend in the number of teenagers engaging in deliberate substance abuse, with a corresponding rise in associated fatalities (13).

First, regarding the characteristics of deliberate substance abuse, our findings from the period spanning 2020 to 2022 substantiate this upward trajectory, with a notably higher incidence among female teenagers compared to their male counterparts, aligning with multiple domestic and international research outcomes (14, 15). While previous research has suggested various explanations for this gender difference, our study design does not permit causal inferences about the underlying mechanisms. The types of medications involved in deliberate substance abuse among teenagers are diverse (16, 17), with sedatives, antipsychotics, and antidepressants being the most commonly misused substances. Our study reveals a predominant misuse of antipsychotic drugs among the adolescents in our clinical sample, with a significant number also resorting to pesticides. The high utilization rate of antipsychotics may be attributed to their accessibility and prevalence (18). The relative ease of obtaining pesticides in China, an agricultural powerhouse, accounts for the observed discrepancy in the types of drugs reported in this study compared to international reports (19).Our findings regarding the predominance of antipsychotic medications in deliberate substance abuse are consistent with previous reports from China (10), though they differ from Western studies where analgesics and sedatives are more commonly involved (15). This discrepancy may reflect regional differences in medication accessibility and prescribing patterns.

Second, regarding associated factors, our correlation analyses indicated significant relationships between adolescent psychological states and factors such as the presence of medical personnel in the family, family structure, guardian’s education background, residential area, sole child status, knowledge of pharmaceutical security, melancholy/uneasiness, CERQ-C scores, adverse experiences, and social support. We postulate that the majority of patients engaging in deliberate substance abuse grapple with mental well-being challenges like diminished self-worth and fluctuating emotions (20). Furthermore, the familial setting holds considerable sway in affecting adolescent psychology, with adverse family conditions such as family conflicts, single-parent families, low educational level of guardians, and poor family economic status negatively impacting adolescents negatively (21).

Third, our multivariate analysis highlights guardian’s education background, knowledge of pharmaceutical security, presence of melancholy/uneasiness, CERQ-C scores and social support as the primary influencing factors on the psychological states of adolescents engaging in deliberate substance abuse. It is important to note that these are correlational findings, and causal interpretations should be avoided. The complex interplay between these factors warrants further investigation through longitudinal designs.

Based on our findings, we recommend enhancing the dissemination of knowledge pertaining to deliberate substance abuse among guardians and adolescents, preventing deliberate substance abuse at the family level. It is crucial to focus on educating adolescents about medication use and emphasizing the importance of strict management of household medications (22). Attention should also be paid to individual psychological health issues among adolescents, offering prompt mental health guidance and corrective actions to strengthen their resilience. Additionally, focusing on the impact of family and social environments and enhancing social support are crucial in creating a constructive and nourishing developmental setting for adolescents (23).

This study provides a detailed characterization of deliberate substance abuse in a clinical adolescent sample, utilizing multiple validated instruments to assess psychological correlates. However, several limitations should be noted. The retrospective design and relatively small sample size from a single clinical setting may affect generalizability. The cross-sectional nature of the data prevents causal inferences. Additionally, we focused on RBQ-A total scores rather than subscale analyses, which may obscure nuances in specific risk behavior domains. The lack of long-term follow-up limits our understanding of the long-term outcomes of these adolescents. Future research should include prospective designs with larger, more diverse samples, longitudinal follow-up, and subscale-level analyses to better understand the trajectories and outcomes of adolescents who engage in deliberate substance abuse.

Our findings highlight several modifiable factors that could be targeted in prevention efforts, including improving medication safety education for adolescents and their families, enhancing social support systems, and implementing routine screening for depression and anxiety in clinical settings. Family-based interventions that address guardian education and family dynamics may be particularly beneficial in reducing deliberate substance abuse among adolescents.

In conclusion, deliberate substance abuse among adolescents constitutes a complex psychological issue, with its related risky behaviors being multifaceted and requiring multidisciplinary research and multilevel intervention measures. By delving deeper into the traits of these behaviors and their psychological states, enhanced preventive and intervention tactics may be developed to curtail the occurrence of deliberate substance abuse among adolescents.

## Data Availability

The original contributions presented in the study are included in the article/supplementary material. Further inquiries can be directed to the corresponding author.
